# Dynamic Adaptation Attack Detection Model for a Distributed Multi-Access Edge Computing Smart City

**DOI:** 10.3390/s23167135

**Published:** 2023-08-12

**Authors:** Nouf Saeed Alotaibi, Hassan Ibrahim Ahmed, Samah Osama M. Kamel

**Affiliations:** 1 Computer Science Department, Shaqra University, Dawadmi City 11911, Saudi Arabia; 2 Informatics Department, Electronic Research Institute, Cairo 12622, Egypt; hassanibrsayed@eri.sci.eg (H.I.A.); samah@eri.sci.eg (S.O.M.K.)

**Keywords:** internet of things, smart city, multi-access edge computing, intelligent process automation (IPA), intrusion detection system (IDS), Random Forest Trees, k-Nearest Neighbor, Bagging, AdaBoost, deep reinforcement learning

## Abstract

The internet of things (IoT) technology presents an intelligent way to improve our lives and contributes to many fields such as industry, communications, agriculture, etc. Unfortunately, IoT networks are exposed to many attacks that may destroy the entire network and consume network resources. This paper aims to propose intelligent process automation and an auto-configured intelligent automation detection model (IADM) to detect and prevent malicious network traffic and behaviors/events at distributed multi-access edge computing in an IoT-based smart city. The proposed model consists of two phases. The first phase relies on the intelligent process automation (IPA) technique and contains five modules named, specifically, dataset collection and pre-processing module, intelligent automation detection module, analysis module, detection rules and action module, and database module. In the first phase, each module composes an intelligent connecting module to give feedback reports about each module and send information to the next modules. Therefore, any change in each process can be easily detected and labeled as an intrusion. The intelligent connection module (ICM) may reduce the search time, increase the speed, and increase the security level. The second phase is the dynamic adaptation of the attack detection model based on reinforcement one-shot learning. The first phase is based on a multi-classification technique using Random Forest Trees (RFT), k-Nearest Neighbor (K-NN), J48, AdaBoost, and Bagging. The second phase can learn the new changed behaviors based on reinforced learning to detect zero-day attacks and malicious events in IoT-based smart cities. The experiments are implemented using a UNSW-NB 15 dataset. The proposed model achieves high accuracy rates using RFT, K-NN, and AdaBoost of approximately 98.8%. It is noted that the accuracy rate of the J48 classifier achieves 85.51%, which is lower than the others. Subsequently, the accuracy rates of AdaBoost and Bagging based on J48 are 98.9% and 91.41%, respectively. Additionally, the error rates of RFT, K-NN, and AdaBoost are very low. Similarly, the proposed model achieves high precision, recall, and F1-measure high rates using RFT, K-NN, AdaBoost, and Bagging. The second phase depends on creating an auto-adaptive model through the dynamic adaptation of the attack detection model based on reinforcement one-shot learning using a small number of instances to conserve the memory of any smart device in an IoT network. The proposed auto-adaptive model may reduce false rates of reporting by the intrusion detection system (IDS). It can detect any change in the behaviors of smart devices quickly and easily. The IADM can improve the performance rates for IDS by maintaining the memory consumption, time consumption, and speed of the detection process.

## 1. Introduction

IoT in the current era presents an essential shift in the way billions of devices and people communicate and exchange vast amounts of information. One of IoT’s applications is a smart city that provides an intelligent and comfortable environment. There are many different domains of smart cities, such as healthcare, smart industries, smart homes, smart resource management, smart building, smart lighting, smart shopping, environmental pollution, and smart security systems. The smart city makes an important contribution to the healthcare domain by facilitating medical operations and follow-up between patients and medical staff. The smart city decreases costs and uses remote monitoring to manage services [1].

**Figure 1:**
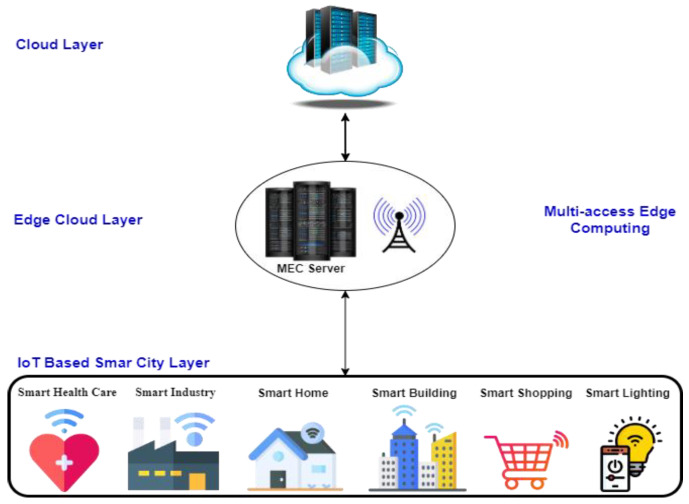
IoT-based smart city architecture.

The structure of an IoT-based smart city comprises three layers, which are named the IoT-based smart city layer, the edge cloud layer, and the cloud layer (as shown in Figure 1). The IoT-based smart city layer contains many applications that yield a huge amount of data. The generated dataset is collected, processed, transferred, and exchanged between the IoT-based smart city and the edge cloud layer. The edge cloud layer is based on a fifth-generation (5G) mobile network. 5G is a new communication technology that provides high speed, high bandwidth, high availability, low latency, and massive connectivity to obtain an intelligent IoT-based smart city. The edge computing system is the main element of 5G, which supports real-time applications. The most common type of edge computing is multi-access edge computing (MEC), which improves power consumption, service capabilities, and reduces cost [2]. MEC can transfer a huge amount of generated data to the cloud. MEC can reduce power consumption and latency. MEC is a centralized application running in real time for the end-users to provide a fast response to the smart city [3]. The data from the edge layer can be collected and then sent to the cloud layer, which is responsible for the storage, analysis, processing, and computation of complex data. Therefore, all users can access data and applications from the cloud layer easily. For this reason, there are many different threats and attacks in MEC in the edge cloud layer.

As a result of the nature of such a heterogeneous IoT network, IoT networks are exposed to many different types of attacks. Subsequently, smart city infrastructure opens different gaps between threats and attacks. An improvement in the security level concept is to achieve security requirements and save power consumption in smart devices. The most common security approach is an IDS, which is used to identify malicious events/behavior of network traffic and attacks. IDS proves the services available in the smart city environment, as it prevents attacks that make the system vulnerable and exhausted. It provides more information about the activities of hosts and has low complexity. Network-based IDS (NIDS) can detect unknown behaviors in network traffic and monitor a large network.

Intrusion detection methods are variously classified as signature-based methods, specification-based methods, anomaly-based methods, and hybrid methods. In the case of the signature-based method, it can analyze data in the network and store it in a database. If it detects new events, it will compare them with the stored data in the signature database [4]. The advantage of signature-based methods is that they are more accurate than other intrusion detection methods. The system administrators adjust the group of rules and thresholds in specification-based methods to evaluate the state of the network. If a given set of rules is broken and the threshold exceeds the limit, then the IDS will detect abnormal events. With regard to the anomaly-based method, it can identify unknown and abnormal network traffic and then compare it with traffic patterns. The disadvantage of this method is that it gives high false positive rates. As regards the hybrid method, it is a combination of signature-based and anomaly-based methods to overcome the disadvantages of existing detection methods. This method can improve the performance metrics of IoT networks [4].

On the other hand, traditional IDS systems cannot detect attacks effectively. Thus, attackers can expose all smart devices to obtain more information about users’ locations as well as their private information. Moreover, attackers can damage the entire network and consume all network resources. Therefore, it should build an intelligent training and testing model to detect and predict such attacks and threats with high performance rates, low time consumption, and high speed. A novel development automation technique is IPA, which can learn from historical events or network traffic and detect and predict unknown events and network traffic with a high prediction rate. IPA is a fast technique with highly accurate predictions and a low error rate. IPA can reduce costs and time consumption while increasing the system’s speed to obtain more flexibility and thereby improve the quality of the learning model. IPA improves End-to-End Process Automation [5]. Therefore, IPA can improve upon the principle of traditional IDS by increasing its performance rate and making it faster. IPA combines two techniques, namely Robotic Process Automation (RPA) and artificial intelligence (AI). RPA can emulate human actions to capture data, run applications, and trigger responses to implement a set of simple tasks. AI encompasses a wide domain of technologies and techniques to allow machines to execute many actions such as decision-making, image recognition, anomaly detection, etc. [5].

The proposed model is based on using NIDS for an anomaly-based detection method based on IPA. Moreover, the proposed model is used to improve the speed of NIDS for anomaly-based detection methods and increase high prediction rates, low error, and low time consumption. Therefore, IPA can improve the quality of the learning model. The proposed model consists of two phases. The first phase contains five modules, specifically a dataset collection and associated pre-processing module, an intelligent automation detection module, an analysis module, a detection rules and action module, and a database module. Each module includes an ICM, which contains all the updated information. The target of ICM is to create feedback reports about each module to send all the information to the subsequent modules. Subsequently, ICM can manage and store all the copies of each module. Any change in each process can be readily detected and labeled as an intrusion. ICM may reduce the search time, increase the speed, and increase the security level.

A proposed model is based on using different classification algorithms such as RFT, k-NN, J48, AdaBoost (J48 is the base learner), and Bagging (J48 is the base learner). RFT, K-NN, Bagging, and AdaBoost give high performance metrics with low errors and increase the speed of the model. The RFT classifier reduces the over-fitting problem and helps improve accuracy. RFT is more flexible and works in both classification and regression. The K-NN classifier is a robust learning algorithm that gives high performance rates. J48 gives lower performance metrics than RFT and K-NN. Therefore, AdaBoost and Bagging are used to improve J48 performance rates. AdaBoost is the common strong ensemble learning algorithm that makes weak learners work better than a single classifier. Regarding the Bagging ensemble learning algorithm, this makes the model more reliable in its predictions to achieve a stable and robust model. Additionally, AdaBoost and Bagging may prevent the over-fitting problem.

This article presents two archived contributions:

1. A proposed model (IADM) produces model verification to detect and predict attacks using multi-class classification with high performance rates. Consider an intrusion detection model that is used to identify online threats. When a zero-day assault occurs on the system, the IDS repeatedly transmits the session records to a cybersecurity administrator to investigate the behaviors of users. The cybersecurity administrator marks the records as malicious or normal activities. This leads to introducing a false positive rate. Additionally, when the limits between the observation of normal and abnormal behaviors are not clear, normal activity changes quickly, which is difficult to learn offline.
2. IADAM produces the detection model, which is used to prove this ability through the dynamic adaptation of the attack detection model based on reinforcement one-shot learning. The proposed model has the ability to autoconfigure and render any changed behaviors associated with the smart city environment as outdated. Autoconfiguration reduces false decisions in the system as it makes the system aware of zero-day attacks. Further, the model is adapted to the changed strategies based on a low number of instances. The reinforce learning method is used in the model to enable it to adapt to new and changed instances. One-shot learning is integrated into the model to create a comprehensive approach to a real-world system for categorizing major events from a limited number of training instances using computational power with constrained resources. Therefore, IADAM can use one-shot learning to achieve auto-adaptation rapidly using a lower number of instances.

Many different IDS techniques, especially the NIDS approach, were based on the UNSW-NB15 dataset, which contains various kinds of attack scenarios to detect malicious traffic. Whereas DARPA98, KDDCUP99, and NSL-KDD have a limited number of attacks.

The transcript is organized into the following sections: Section 2 provides a discussion and a literature review, whereas Section 3 presents the methodology of a proposed model, which explains the system model and the proposed model as integrated with the IoT-based smart city, and Section 4 presents the evaluation and experimental results. Section 5 finally offers concluding remarks.

## 2. Literature Review

In the literature, much research has been presented that seeks to enhance IDS performance rates. We highlight more recent research based on deep learning and machine learning techniques to achieve higher performance rates. From the previous studies, it is evident that most of these studies relied on improving the performance rates of IDS without considering memory consumption, power consumption, or resource consumption across IoT networks.

Rashid et al. (2020) [1] proposed a model to protect the IoT smart city from attacks using different machine learning algorithms such as Logistic Regression (LR), Support Vector Machines (SVMs), Decision Tree (DT), Random Forest (RF), Artificial Neural Networks (ANNs), k-Nearest Neighbors (k-NNs), Bagging, Boosting, and Stacking. One proposed model is based on anomaly detection techniques to enhance the performance rates of the detection system. All machine learning algorithms, including ensemble learners, were based on a multi-class classification of attacks. The UNSW-NB15 and CICIDS2017 datasets were used to introduce different types of attacks. The experimental results showed that the ensemble learner gave better performance metrics than the individual algorithms in terms of accuracy, precision, recall, and F1-score. This led to the conclusion that ensemble learning algorithms were more robust than individual algorithms. Although the ensemble learning algorithms were robust, they consumed a great deal of time to detect IoT smart city attacks. These experiments were implemented using the Python programming language.

Hasan et al. (2019) [6] introduced a comparative study of different machine learning models, specifically LR, SVM, DT, RF, and ANN, to predict attacks in IoT networks. This work was based on a Distributed Smart Space Orchestration System (DS2OS) dataset, which consisted of 357,952 (347,935 normal and 10,017 abnormal) records with 8 classes and 13 features. The experiments achieved a high degree of accuracy for DT, RF, and ANN, approaching 99.4%. Additionally, the detection rate of the Denial of Service (DoS) attack was higher than with other approaches. The experimental results proved that RF detected most IoT network attacks with a high accuracy rate. This study was implemented using Pandas and Numpy frameworks for data analogy, cleaning, and feature engineering, whereas data visualization was implemented using Matplotlib and Seaborn frameworks. The Scikit-learn framework and Keras framework were used for the data analysis.

C. Liang et al. (2020) [4] proposed a system that was designed to implement and test an IDS using a hybrid placement strategy that was based on a multi-module system, which was itself based on blockchain using deep learning algorithms. The purpose of using blockchain was to secure the system from different types of attacks, information tampering, and information disclosure. The proposed system could be distributed to various remote hosts. The multi-module system was based on a reinforcement algorithm to continuously enhance the performance of the system. The proposed system consisted of four modules, namely data collection, data management, analysis, and response. The NSL-KDD dataset was used in this work. The experimental results showed that deep learning algorithms could detect many attacks on the transport layer in an IoT network. The experiments explained that the proposed model achieved higher performance rates in different scenarios, such as network complexity and different types of attacks. This research aimed to achieve high performance metrics using the application of distributed AI to simplify a complex network. The proposed system achieved high performance metrics on a test set, with accuracy, precision, recall, and F-scores of 83.9%, 85.53%, 84.14%, and 83.94%, respectively. The proposed system was implemented using SESS (foundation of the intelligent physical agent–agent communication language FIPA-ACL), and the detection methods used Java classes.

Alrashdi et al. (2019) [7] proposed an anomaly detection-IoT (AD-IoT) system based on an RF. The proposed model was implemented to detect malicious behavior before distributing fog nodes at the cloud layer, employing a UNSW-NB-15 dataset. The proposed model achieved a high degree of accuracy (99.34%), a low false positive rate (2%), and a high detection rate (82%). The proposed model was implemented using the Python programming language.

Anthi et al. (2019) [8] proposed three layers of an IDS to detect attacks in IoT-based smart homes. The proposed system comprised three core functions. The first core function classified the normal behavior of each IoT device based on their MAC addresses. The second core function identified malicious packets from IoT devices as being benign or malicious. The third core function classified malicious packets according to the nature of the IoT network attacks. The proposed system could detect many attacks such as DoS, Man-In-The-Middle (MITM)/spoofing, Reconnaissance, and Replay. The experiments were implemented using nine machine learning algorithms, which were, specifically, Naïve Bayes, Bayesian Network, J48, Zero R, OneR, Simple Logistic, SVM, Multi-Layer Perceptron, and RF. The experiments were based on the IoT Smart Home Testbed, which consisted of popular camera displays of smart IoT devices. The evaluation metrics were based on using precision, F-measure, and recall. The F-measure results from the three core functions were 96.2%, 90.0%, and 98.0%, respectively. The proposed model was implemented using the open-source software Weka.

Al-Haija and S. Zein-Sabatto (2020) [9] introduced an intelligent and autonomous deep learning approach (IoT-IDCS-CNN) to detect many attacks across IoT networks. The proposed model was based on high-performance computing and parallel processing. The proposed system consisted of three subsystems, namely a featured engineering subsystem, a featured learning subsystem, and a traffic classification subsystem. The NSL-KDD dataset was used in this research, and the experimental results demonstrated a high degree of accuracy of the binary and multi-class classifications of 99.3% and 98.2%, respectively. Additionally, the experimental results achieved high precision, recall, F1-score, and a low false alarm rate. The proposed system was implemented in MATLAB 2019a.

Abeshu and Chilamkurti (2018) [10] introduced a novel model using distributed deep learning to detect cyber-attacks in fog computing. The experiment was based on the usage traffic distribution of the NSL-KDD dataset. The deep learning model achieved 99.20% accuracy, 99.27% DR, and 0.85% FAR. In contrast, the shallow learning model achieved 95.22% accuracy, 97.50% DR, and 6.57% FAR. Therefore, the proposed model based on deep learning achieved greater accuracy than the shallow model. The experiments were implemented using DL package Keras on Theano and Apache Spark for the distributed processing framework.

N. Koroniotis (2017) [11] discussed machine learning techniques to identify and track network traffic by detecting botnet activities in a network forensic mechanism. The experiments were implemented using C4.5, NB, Association Rule Mining (ARM), and Artificial Neural Network (ANN). The proposed model was based on the UNSW-NB15 dataset. The experimental results revealed that the C4.5 classifier achieved a higher accuracy of 93.23% and a lower FAR at 6.77%. The C4.5 classifier was the best at differentiating between botnet attacks and normal records in the network traffic. The proposed model was implemented using the open-source software Weka.

Nawir et al. (2018) [12] introduced a proposed model based on an Online Average One Dependence Estimator (AODE) algorithm using a multi-classification method. The experiments were performed using the UNSW-NB15 dataset. The proposed model may solve the problem of complex, dynamical, and streaming data in a network system. The proposed model can detect network attacks with a high degree of accuracy, 99.93%, and a low false positive rate (FPR) of 0.001%. In comparison, online Naïve Bayes (NB) achieved an accuracy of 69.60%, where the classification rates were 83.47% and 69.60%. However, the online NB model is faster than the online AODE model. The proposed model was implemented using Ubuntu, software version 13.10-0 Ubuntu 4.1, and open-source software WEKA (WEKA 3.8).

Hassan I. Ahmed et al. (2021) [13] introduced DADEM, which is a distributed deep learning model that uses a big data analytics platform for distributed processing to create an AEASGD training approach. DADEM combined two techniques named deep learning and big data analytics. The sequential deep learning model was chosen as a classification engine for the distributed processing model. The obtained results were compared with other classification algorithms like logistic regression, KNN, ID3 decision tree, CART, and SVM. The experimental results showed that the sequential deep learning model outperformed the ones mentioned above. The proposed model achieved high accuracy, achieving 99.64% and 99.98% for the UNSW-NB15 and BoT-IoT datasets, respectively. Moreover, the proposed model can reduce the overhead of the overall system operation in IoT.

Hassan I. Ahmed et al. (2022) [14] presented the Cross-Layer Distributed Attack Detection model (CLDAD) to improve security solutions for the IoT environment. CLDAD presented a general detection method of DDoS in the sensing layer, network layer, and application layer based on big data analytics techniques. This can enable the detection process in a distributed way to detect DDoS attacks in any layer on-the-fly. Therefore, the proposed model can support the scalability of the IoT environment. The experiments were tested based on three datasets, namely, the artificial jamming attack dataset, the BoT-IoT dataset, and BoT-IoT-based HTTP. The experimental results showed that the proposed model was effective for detection attacks in the three layers. CLDAD achieved a high detection accuracy of 99.8% on average.

The gap analysis of the IADM-based one-shot learning and other proposed models in the detection and classification of network attacks are summarized in Table 1. The contribution of the IADM framework is its ability to adapt to the environment, which is a better-known property of IoT. Scalability and changed behaviors are the most widely known adjectives of IoT networks. The IoT environment is considered a fast-changing one due to the constant addition of new sensors, users, devices, and applications, etc. The rapid variant detection model can be updated through reinforcement learning, which requires relatively few samples or instances.

## 3. Methodology

**Figure 2:**
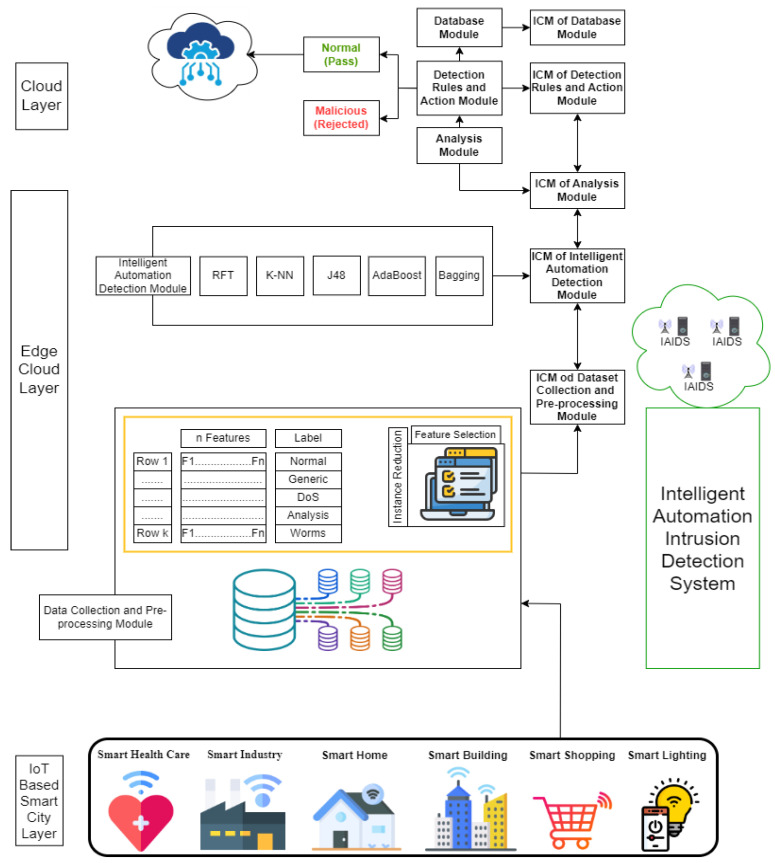
IoT-based smart city framework using intelligent automation detection model (IADM).

The proposed model is implemented to detect and prevent unknown network traffic attacks, malicious behaviors, or abnormal events at distributed MECs in the IoT-based smart city with the ability to auto-adapt to changed or novel behaviors. The model is based on the IPA technique and the NIDS for anomaly-based detection methods. The model can monitor network traffic and events that pass through each distributed MEC to detect and predict attacks and threats. The learning process relies on learning from historical events/network traffic to detect and predict unknown input network traffic and events accurately. The model consists of two phases. The first phase comprises five modules that are named dataset collection and pre-processing, intelligent automation detection, analysis, detection rules and action, and database, respectively. Each module contains an intelligent unit named ICM, which contains all the updated information. ICM can create feedback reports for each module that are sent to the next ICM in the adjacent module to manage and detect any changes in the proposed model. At the same time, ICM can store a copy of the feedback reports for each phase of the proposed model. Finally, the results are stored in the database module. When any change in feedback reports is noticed, it will be detected and labeled as an intrusion. Therefore, ICM can investigate the dataset to detect different types of attacks. Consequently, it is difficult for any attacker to penetrate the IoT network at any stage. The ICM may reduce search times, increase the speed, and increase the levels of security. The second phase depends on one-shot learning using a dynamic adaptation of the attack detection model based on the reinforcement learning model. The second phase aims to convey the ability to auto-adapt to changed or novel behaviors. Moreover, it can detect zero-day attacks and any unexpected changes in the IoT network. The IoT-based smart city framework is based on using the IADM, which is illustrated in Figure 2.

### 3.1. System Design

**Figure 3:**
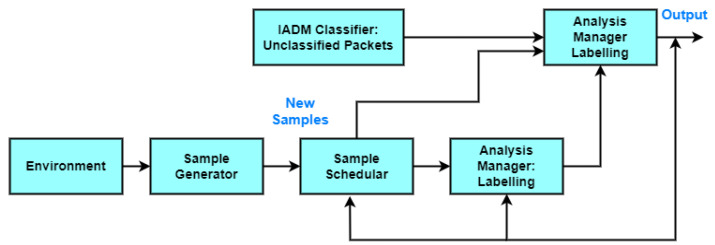
Auto-adaptive IADM based on ROL.

The system design is built using two phases. The first phase is an intelligent automation IDS (IADM). The idea of the first phase is based on the IoT-based smart city network framework. Every module generates and processes the generated data, which are transferred to the next module. The framework is based on using 5G to communicate many smart devices to others with high bandwidth and low latency. The main element in 5G is the distributed MEC that is used to exchange data, which is transferred to the cloud layer. Each distributed MEC uses an intelligent automation IDS (IADM) to detect and track the network traffic and events that move from/to each MEC and end-users automatically. IADM will be more functional for the detection and prediction of network traffic attacks. After detecting attacks, data can be safely transferred to the cloud layer. The second phase is based on one-shot learning using a dynamic adaptation of the attack detection model based on the reinforcement learning model to detect zero-day attacks and unexpected changes in the IoT network, as shown in Figure 3.

Figure 3 represents the unclassified packets of the IADM where the newly changed behaviors are the inputs of the reinforced one-shot learning model. The changes in the IoT network are sampled and prepared as a new instance for the reinforcement one-shot learning (ROL) model. A few numbers of instances are the input of the ROL model. The ROL model can classify a small number of training instances. The output of the ROL model constitutes some of the newly adapted feature weights for the IADM.

### 3.2. First Phase: An IADM Design Model Integrated within the IoT-Based Smart City

**Figure 4:**
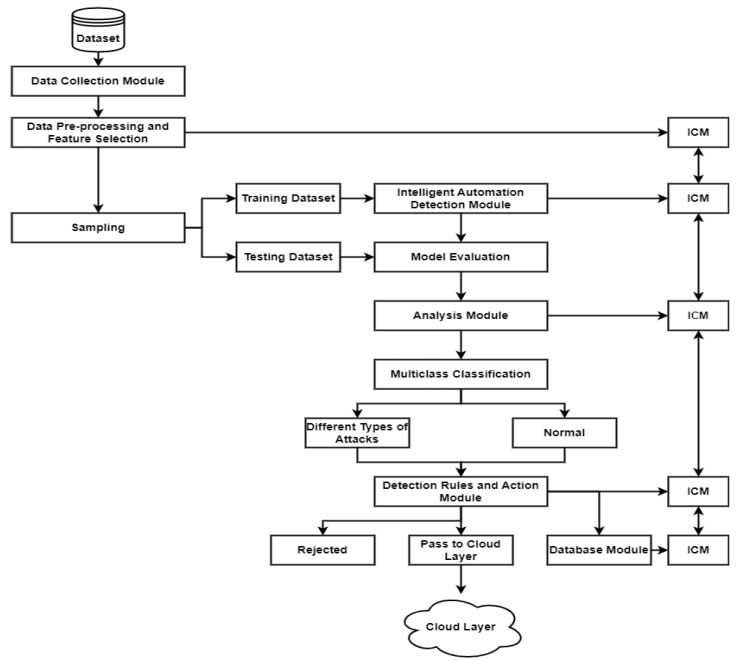
The process underlying an intelligent automation IDS (IADM).

IAMD can monitor and manage IoT network traffic and any events through a distributed MEC to detect unknown attacks. Datasets can be collected from the dataset collection module and pre-processed. The feature selection method is used to select the most significant features. The training dataset can be taught using an intelligent automation detection module. The testing dataset is evaluated using model evaluation. An analysis module can be used to analyze all the events and the network traffic. IAMD is based on a multi-class classification technique using different classification algorithms to differentiate between different kinds of attacks and normalize the network traffic and events. A detection rule and action module can decide to reject all malicious network traffic/events or pass the normal traffic/events onto the cloud layer. All the results can be stored in a database module. Each module has an ICM unit to create feedback reports about each module to facilitate detection attacks with high performance rates that consume less system time. Additionally, ICM can increase the speed of storage usage data in each module. Figure 4 illustrates the process of the proposed model.

#### 3.2.1. Dataset Collection and Pre-Processing Module

The dataset collection module collects datasets from multiple sources and stores all the data from the smart city. The dataset pre-processing process is a very important step to pick out the most significant features to improve performance rates. Pearson’s correlation coefficient, or Pearson’s R, is a common approach to the feature selection method. Then, the ICM creates a feedback report about the dataset collection and pre-processing module. Additionally, ICM can save a copy of the feedback report within this module. The feedback report will be sent to the ICM of the intelligent automation detection module.

#### 3.2.2. Intelligent Automation Detection Module

The ICM feedback reports from the dataset collection and the pre-processing module are sent to the ICM of the intelligent automation detection module, which detects a new dataset. The intelligent automation detection module is based on the learning process in order to distinguish between normal and malicious activities. In this module, the intelligent automation detection module receives network traffic and events (dataset) from ICM using the RFT, k-Nearest Neighbor (k-NN), J48, AdaBoost (J48 is the base learner), and Bagging (J48 is the base learner) models to analyze and detect any anomalous behaviors. When this module receives any unknown dataset, it will analyze the behavior of anomalous network traffic and events and produce a testing model along with its evaluation. The results will be sent and stored in the ICM, which will create a feedback report that will be sent to the ICM of the analysis module.

#### 3.2.3. Analysis Module

In the analysis module, the feedback reports from ICM of the intelligent automation detection and analysis modules are used to analyze and extract all the events and network traffic patterns from multiple sources. This provides a vision of all the events and network traffic. When the system receives an anomalous dataset, it will compare the unknown dataset with historical events or records to detect malicious events and behaviors. The results will be stored in ICM and produce a feedback report, which will be sent to the ICM of the detection rules and action module.

#### 3.2.4. Detection Rules and Action Module

In the detection rules and actions module, the IPA checks the updated dataset from the dataset collection and pre-processing module. The updated dataset then compares it with the historical dataset and checks the management ruleset.

**Figure 5:**
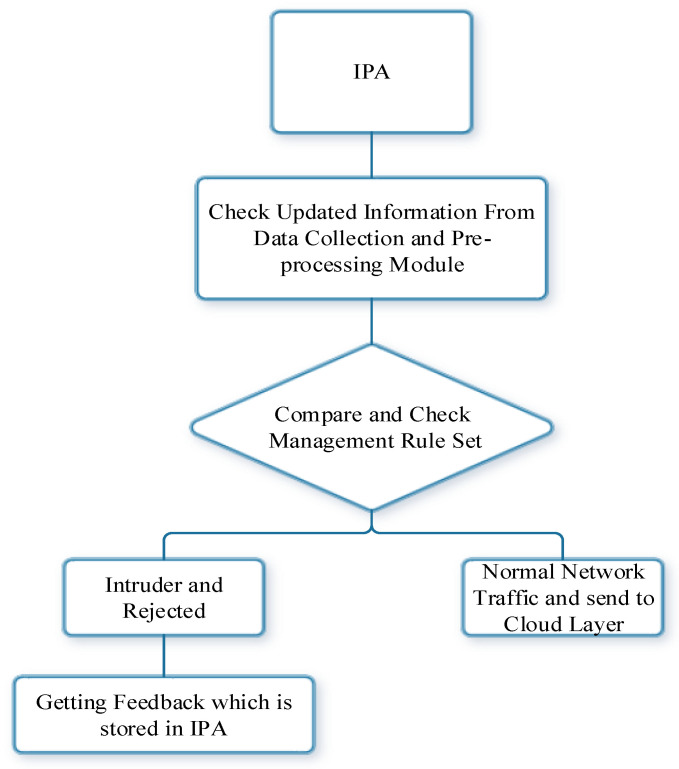
Flowchart of detection rules and action modules.

As mentioned above, in the proposed model, the management ruleset includes the rule of IF-THEN. IPA will check the updated and uploaded network traffic that comes from data collection and the preprocessing module. The obtained result will be compared with the history of network traffic. If the results are normal, network traffic and events will be sent to the cloud layer. If the obtained result is abnormal network traffic, then it will be labeled as an attack and will be rejected. This module will then provide a feedback report that will be stored in the ICM. This report will be sent to the database module as per the following schematic (Figure 5).

#### 3.2.5. Database Module

The responsibility of the database module is to alter the database according to the feedback reports of the ICM. When the database module receives the updated information from the ICM of the detection rules and action module (according to a new dataset), then the database module changes the database and sends a feedback report to the ICM. Therefore, the ICM can store the copies from each module to record any change in the IoT-based smart city networks to block any threat and/or attack. Additionally, the ICM may decrease the response time and increase the speed of intelligent detection.

### 3.3. The Second Phase: Dynamic Adaptation of the Attack Detection Model Based on the Reinforcement of One-Shot Learning

The problems concerning IADM are that zero-day attacks and unexpected changes in the environment cause classification problems in the detection model. As a result, the likelihood of a false positive or false negative will be increased. It is difficult to use traditional machine learning or deep learning because they need to be trained with a large number of instances to achieve good classification accuracy. To overcome these problems, the proposed model presents a convenient solution, which is illustrated as follows. Training the model based on a small number of instances is considered “one-shot learning”. Reinforcement learning can deal with a small number of state spaces and is thus impractical for the problem of large-size instances because it utilizes a lookup table for classification, which needs memory that degrades performance when the state space increases. Reinforcement learning/one-shot learning is suitable for the learning process. The detection model for the new changes within the IoT environment changes rapidly, and it has only limited resources.

The optimization plan of the detection model is used to detect zero-day attacks and to be dynamically adapted to changes within the IoT network. Additionally, it increases the accuracy and reduces the false positive and false negative rates of the classifier. This can be implemented by learning the model over a small number of training instances within the IoT network. The training instances can be collected from the IoT network, and these training samples can be generated by a sample generator and then labeled manually via analysis. The training samples are input into a ROL model as new instances.

Reinforcement learning, or Q-learning, can be performed with a small number of instances but cannot deal with a large number of instances. Reinforcement learning, or Q-learning, is used for storing the search table of the long-term database for all possible state-action pairs. This makes complexity within the storage process a major problem.

The second is the low efficiency of the training process in many states. If it is necessary to train the model for a large number of instances, then reinforcement learning can be combined with deep learning (otherwise referred to as a Deep Q-Network (DQN) or deep reinforcement learning (DeRL)).

Assuming that there are changes in the IoT environment, one-shot classes will arrive and need to be classified. This leads to a need to classify new classes with only a few training instances. The terms can be expressed as follows:

*K* is a possible class

*M* represents human analysts

(*t*) is an action function

*S*(*t*) is a state function of the observed system

(*t*) is the reward

The following equation of Q-learning with a Q-value at each time for each change:

(1) $$Q_{t+1\;}\left(s\left(t\right),\;a\left(t\right)\right)=Q_{t}\left(s\left(t\right),\;a\left(t\right)\right)+\alpha \;[r\left(t+1\right)+\gamma Q_{t}\;\left(s\left(t+1\right),\;a\;\left(t+1\right)\right)-Q_{t}(s\left(t\right),\;a\left(t\right))]\;$$

where

(2) $$r\left(t+1\right)+\gamma Q_{t}\;(s\left(t+1\right),\;a\left(t+1\right))\;$$

The term $a\;\left(t+1\right)$ defines the learned value obtained, where 𝑡 is an action in state *s*(*t*). The next step defines *s*(*t* + 1). The taken action (*t* + 1), which increases the future *Q*-value seen at the next state in *Qt* (*s*(*t*), *a*(*t*)) is the old learned value. The algorithm aims to decrease the time difference (TD) error between the learned value and the current evaluated value. The following equation describes the loss function that is used for updating the *Q*-values for every training by ROL.

(3) $$\sum_{t}{[Q_{t}\left(s\left(t\right),a\left(t\right)\right)-(r\left(t+1\right)+\gamma \;\underset{\alpha (t+1)}{\max}Q_{t}(s\left(t+1\right),a\left(t+1\right)))]}^{2}$$

The classifier must have one-shot learning capabilities. It is more complex to classify and learn new classes.

## 4. Evaluation and Experimental Results

The forthcoming section presents an evaluation of the proposed model using the UNSW-NB15 dataset. This study was evaluated using RFT, K-NN, J48, AdaBoost, and Bagging to build learning and testing models so as to identify normal and malicious network traffic, behaviors, and events at a distributed MEC within the IoT-based smart city during the first phase. Reinforcement learning occurs in the second phase to detect new changes in the smart city given constrained resources.

### 4.1. Datasets

Older datasets such as KDDCUP 99 and NSL-KDD are inadequate for the detection of modern attacks because they have redundant instances and missing values. In 2015, the IXIA Perfect Storm tool was used to create a modern hybrid dataset, UNSW-NB15, that contains both normal and abnormal network traffic [15]. The period of creation of UNSW-NB15 was around 15 to 16 h over two days. The UNSW-NB15 dataset contains new attacks from captured network traffic. The UNSW-NB15 dataset includes 48 features, namely srcip, dstip, dsport, proto, state, dur, sbytes, dbytes, sttl, dttl, sloss, dloss, service, Sload, Dload, Spkts, Dpkts, swin, dwin, stcpb, dtcpb, smeansz, dmeansz, trans_depth, res_bdy_len, Sjit, Djit, Stime, Ltime, Sintpkt, Dintpkt, tcprtt, synack, ackdat, is_sm_ips_ports, ct_state_ttl, ct_flw_http_mthd, is_ftp_login, ct_ftp_cmd, ct_srv_src, ct_srv_dst, ct_dst_ltm, ct_src_ltm, ct_src_dport_ltm, ct_dst_sport_ltm, ct_dst_src_ltm, and attack_cat. The training dataset contains 175,341 records, whereas the testing dataset includes 82,333 records. The UNSW-NB15 dataset contains both normal instances and nine attacks named generic, exploits, fuzzers, DoS, reconnaissance, analysis, backdoors, shellcode, and worms [16].

### 4.2. Evaluation Criteria

The performance of IAIDS can be estimated using accuracy, precision, recall, and F-measure or F1 score. The determined accuracy is the ratio of the true positive (TP) and TN (correct prediction number) and the total number of predictions. The equation of accuracy can be represented as follows:

(4) $$AC=\frac{TP+TN}{TP+TN+FP+FN}$$

where TP = true positive, TN = true negative, FP = false positive, and FN = false negative.

The following equation defines the classification error, which is the ratio between incorrect predictions and the total number of predictions.

(5) $$Error=\frac{FP+FN}{TP+TN+FP+FN}$$

Precision is the ratio between the TP and the total of both true positive and false positive.

(6) $$Precision=\frac{TP}{TP+FP}$$

Recall is the ratio between the TP and the total number of true positive and FN.

(7) $$Recall=\frac{TP}{TP+FN}$$

$Recall=\frac{TP}{TP+FN}$ F-measure, or F1 score measures the balance between precision and recall.

(8) $$F-measure=2*\frac{Precision*Recall}{Precision+Recall}$$

### 4.3. Experimental Results of Dataset Pre-Processing

The correlation coefficient measures a relationship between two attributes and the strength of this relationship. The most common correlation coefficient is Pearson’s R. The results obtained are −1, 1, and 0, where the value 1 indicates a strong positive relationship. The value −1 indicates a strong negative relationship, and zero indicates no relationship [17]. The Pearson’s R equation can be expressed as follows:

(9) $$r=\frac{n\;(\sum xy)-(\sum x)(\sum y)}{\sqrt{[n\;\sum x^{2}-(\sum {x)}^{2}][n(\sum y^{2})-(\sum {y)}^{2}]}}$$

where *x* is the first feature, *y* is another feature, and n is the sample size.

**Figure 6:**
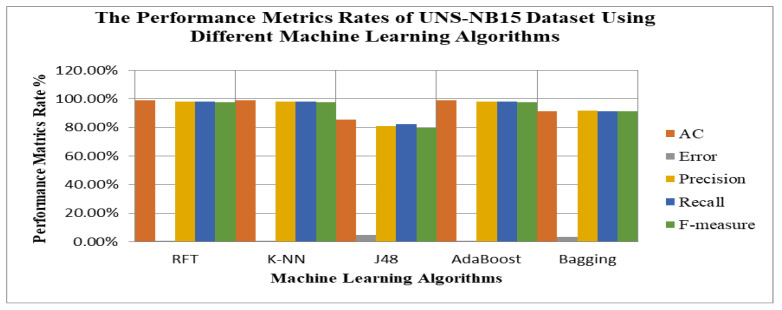
Performance evaluation in terms of accuracy, error, precision, and recall of the UNS-NB15 dataset using different machine learning algorithms.

The feature selection method has been implemented using RapidMiner Studio open-source. The experimental result of the correlation matrix is shown in Figure 6. The correlation coefficient is used to measure the respective features’ weights and the correlation between the features [18,19,20]. The experiment has shown that there are three features of correlation, named positive, negative, and zero. We consider that the correlation ratio of features is greater than or equal to the predetermined threshold of 50%.

The experimental results have shown that the selected features are ct_dst_sport_ltm, ct_src_dport_ltm, ct_dst_src_ltm, ct_srv_dst, ct_srv_src, ct_dst_ltm, Id, is_ftp_login, is_ftp_login, ct_ftp_cmd, service, response_body_len, Sbytes, Rate, Sttl, and label. Table 2 shows the correlation matrix of all the features and gives a form of relationship among all the features.

#### 4.3.1. Experimental Results of the First Phase

Concerning the implementation of phase 1 of the model, the training and test models are built using Weka open-source 3.9.5 [21,22,23,24,25]. The operating system is Windows 10 64-bit, and the processor is an Intel(R) Core (TM) i7-7700 CPU @ 3.60 GHz 3.6 GHz with 16 GB RAM.

The experiments have been implemented using RFT, K-NN, J48, AdaBoost, and Bagging. 10-fold cross-validation (CV) is used where the UNSW-NB15 dataset is randomly divided into 10 equally sized subsets [26,27,28,29]. The experiments are based on applying multi-classification or multi-label using different machine learning algorithms. The accuracy rates (AC) of RFT, K-NN, J48, AdaBoost, and Bagging are 97.99%, 97.99%, 85.51%, 97.99%, and 91.416%, respectively [30,31,32,33,34,35]. According to the results, RFT, K-NN, and AdaBoost models show better results in terms of accuracy than others. Bagging shows a high degree of accuracy, above 90%. In contrast, J48 shows a poor accuracy rate. The performance measures in terms of error for RFT, K-NN, J48, AdaBoost, and Bagging are 2.1%, 2.1%, 14.5%, 2.1%, and 8.6%, respectively. Regarding RFT, K-NN, AdaBoost, and Bagging give lower error rates than the others. On the other hand, J48 has high error rates. Therefore, ensemble learners enhance the performance rate of J48. Additionally, RFT, RFT, K-NN, and AdaBoost achieve a high precision rate of 98%, whereas Bagging gives a precision rate of 91.6%. J48 shows lower precision rates at 80.9%. In terms of recall, RFT, K-NN, J48, AdaBoost, and Bagging achieve 98%, 98%, 82.5%, 98%, and 91.4%, respectively. The recall rates of RFT, K-NN, and Adaboot Bagging yields are better than J48 and Bagging. Finally, the F-measure shows that RFT, K-NN, and AdaBoost achieve higher F-measure rates than either J48 or Bagging. F-measure rates of RFT, K-NN, AdaBoost, J48, and Bagging are 97.5%, 97.5%, 97.59%, 79.5%, and 91.1%, respectively. RFT, K-NN, and AdaBoost outperform other metrics. The performance evaluations of accuracy, error, precision, recall, and F-measure are presented in Table 3 and Figure 6.

The following tables demonstrate the performance metrics of the different classes within multi-class detection using RFT, K-NN, J48, AdaBoost, and Bagging.

From Table 4, the proposed model based on RFT offers high performance rates. The TP rate, precision, recall, and F-measure rates of all the attacks and normal classes are approximately 98%. The proposed model achieves a 2% false positive rate.

Similar to RFT, the proposed model can achieve a high-performance evaluation for multi-class detection using K-NN as shown in Table 5. Th true positive rate (TPR), precision, recall, and F-measure rates of all attacks and normal classes are approximately 98%. The proposed model achieves a 2% FPR.

The proposed model using J48 realizes a lower performance evaluation for multi-class detection than other models. The proposed model using J48 shows its weakness in detecting different types of attacks [36,37,38,39,40,41].

Table 6 presents the performance metrics of different classes in multi-class detection using AdaBoost with the J48 base learner. Table 6 illustrates that the TP rates of analysis, Backdoor, DoS, Reconnaissance, and worm attacks classes are 23.4%, 48.8%, 79%, 77.5%, and 58.1%, respectively. However, the TP rates of exploits, Fuzzers, and shellcode attack classes are 97.4%, 89.9%, and 80.2%, respectively. The FPR of the analysis attack class is higher than for other approaches. The precision rates of exploits and worms attack classes are 67.4% and 58.1%, respectively. The recall rates of analysis and backdoor attack classes are the lowest at 23.4% and 48.8%, respectively. The F-measure rate of the DoS attack class is 14.3%. The J48 classifier is therefore the lowest-performing for the proposed model. As mentioned above, the proposed model uses 10-CV, which increases complexity, and so there is an inherent error in building and splitting trees through the training data. This error leads to the over-fitting problem [42,43,44,45,46,47,48,49]. Therefore, J48 fails to implement multi-classification and gives a low-performance evaluation. To improve the performance metrics of J48, J48 is used as a base learner for AdaBoost and Bagging [50].

From Table 7, AdaBoost can be seen to enhance the performance of the J48 where the TPR, precision, recall, and F-measure results are close to 98%. FPR achieves a 2% rate for all the classes. Thus, AdaBoost improves the performance metrics of J48.

From Table 8, the results show that the analysis attack class offers a lower performance evaluation rates than the other classes, with a high FPR of 13.1%. In terms of the DoS attack class, it gives a lower precision rate of 58.4%. However, Bagging can enhance the performance metrics of the J48 classifier and mitigate the over-fitting problem. AdaBoost with the J48 base learner provides high performance rates because many weak classifiers can work sequentially to improve their performances. AdaBoost improves the performance rates of the single classifier with a higher accuracy rate, lower error, and lower FPR. The proposed model reduces time consumption to build training and test models, which leads to an increase in the speed of the proposed model for attack detection. The previous research did not mention the number of trials required to obtain a minimum time but this experiment has been implemented five times to obtain a minimum time. From Table 9, K-NN and J48 take the least time to implement the test model as compared to AdaBoost and Bagging. On the other hand, AdaBoost and Bagging take a longer time to implement the test model because of the number of AdaBoost and Bagging iterations required.

**Figure 7:**
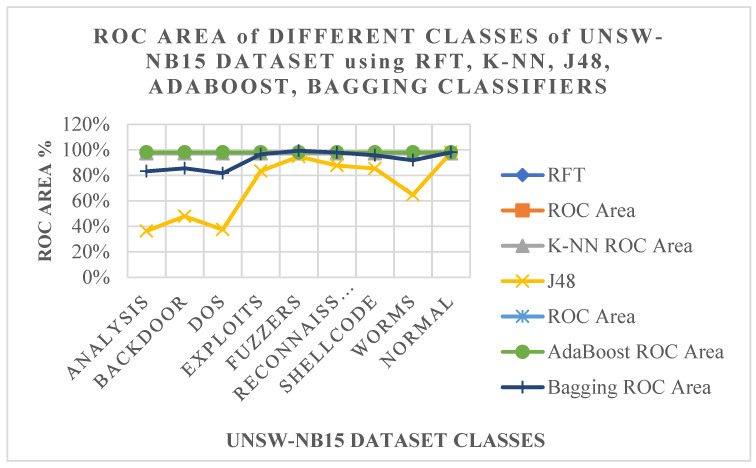
ROC areas using different classes of UNSW-NB15 dataset employing RFT, K-NN, J48, AdaBoost, and Bagging classifiers.

**Figure 8:**
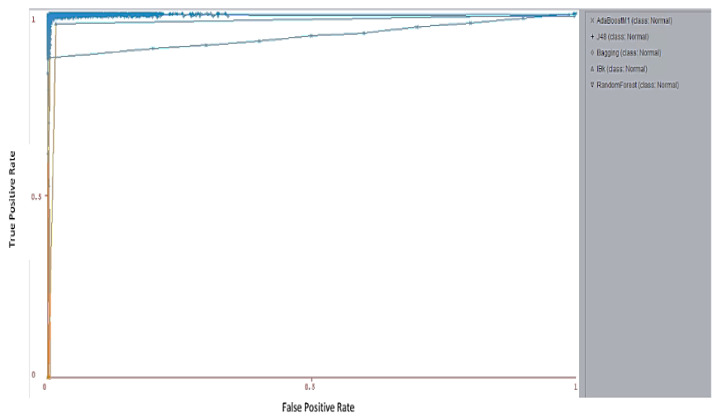
ROC curve of RFT, K-NN, J48, AdaBooat, and Bagging classifiers.

Additionally, an ROC area is a very important factor in demonstrating the probability of attack detection with a given false alarm probability [51,52,53,54,55,56,57]. Figure 7 and Table 10 illustrate the ROC area of different classes of the UNSW-NB15 dataset using RFT, K-NN, J48, AdaBoost, and Bagging. The experimental results show that RFT, K-NN, and AdaBoost offer the best ROC area for all attack and normal classes at 98%. On the other hand, Bagging and J48 achieve 96.6% and 86.6%, respectively. Therefore, Bagging is better than J48. The ROC areas of analysis and DoS classes using J48 are 36.4% and 37.5%, respectively. AdaBoost and Bagging can improve the ROC area of J48. The ROCs for RFT, K-NN, J48, AdaBooat, and Bagging classifiers are shown in Figure 8, which also illustrates the ROCs for RFT, K-NN, J48, AdaBooat, and Bagging classifiers. Finally, our analysis has highlighted the importance of using ensemble learning algorithms.

The ROC curve is the plot of the true positive rate (TPR) against the false positive rate (FPR). In this paper, the ROC curve was used to assess the proposed model, which relies on various thresholds. The ROC curve of RFT, K-NN, J48, AdaBooat, and Bagging classifiers in the following Figure 8. RFT, K-NN, AdaBooat, and Bagging achieved higher TPR based on different attacks and normal classes than the J48.

#### 4.3.2. Experimental Results of the Second Phase

The second phase, named dynamic adaptation, is based on the reinforcement of one-shot learning results. Concerning the challenges of the attack’s detection agent, adapting to changes in the environment and unclassified instances of the detection agent is paramount. The one-shot learning method is used for new and unclassified instances. Network session summary records were categorized in this experiment to differentiate between attacks and normal records/instances [58,59,60,61,62]. This experiment was performed to test the ROL algorithm’s capability for quick attack changes (zero-day attacks). The implementation was done in Python.

Table 11 presents a performance comparison of our model based on ROL. The learning ability of IADM based on ROL is compared with other models such as the Anton model and the Siamese NN [63,64]. The Anton model is a DeROL framework that was developed to solve the classification of a few instances using UNSW-NB15. Siamese NN was developed based on one-shot image recognition to differentiate it from the reinforcement learning expert-based framework, which enhances performance rates [65,66,67,68,69,70,71]. In that part of the classification based on a few training instances, IADM is considered a distinct model based on accuracy and accumulated reward [72,73,74,75,76,77].

Finally, IADM has a comparable accuracy relative to the aforementioned models in the literature reviews. Furthermore, the proposed model can auto-adapt rapidly through one-shot learning and a lower number of instances. IADM is considered more suitable for IoT, which has the advantage of being rapidly changing and scalable.

## 5. Conclusions

IoT technology has become an intelligent way to create a smart life. Despite the IoT introducing a smart solution to providing a more comfortable lifestyle, IoT networks are exposed to many different and various attacks. This paper introduced IPA and an auto-configured IADM to detect and prevent malicious network traffic and behaviors/events at distributed MEC in an IoT-based smart city. The IADM architecture comprised two phases. The first phase relies on using IPA at NIDS, employing an anomaly-based detection method comprised of five modules named dataset collection and pre-processing, intelligent automation detection, analysis, detection rules and action, and database. The first phase relied on an important module named an intelligent connecting module that gave feedback reports about each module and sent information to the subsequent modules. Subsequently, the first phase laid siege to every module in the smart city. ICM assisted in reducing search times, increasing the speed, and increasing the security. The first phase had been implemented using a feature selection mechanism based on correlation coefficients to select the most efficient feature to measure the strength of a relationship between two attributes. The first phase had been implemented using multi-classification algorithms. These, specifically RFT, k-NN, J48, AdaBoost (J48 is the base learner), and Bagging (with J48 as the base learner), were in turn used to build the learning model. The proposed model using RFT, K-NN, and AdaBoost achieved a high accuracy rate of approximately 99%. However, the accuracy rate of the J48 classifier achieved 85.51%, which was less than the others. Subsequently, AdaBoost and Bagging were used to enhance the performance metrics of J48. The accuracy rates of AdaBoost and Bagging were 98% and 91.41%, respectively.

Additionally, this research introduced an auto-adaptive model through the dynamic adaptation of the attack detection model based on ROL. The learning model was built using the reinforcement learning technique because it can use a small number of instances to save memory. Therefore, the auto-adaptive model can be auto-configured and become outdated with any change that occurs in the behaviors of the intelligent devices in the IoT network. Thus, autoconfiguration can reduce the incidence of false rates in the system as it makes the system aware of zero-day attacks. As a result of using one-shot learning, the one-shot learning model is integrated into the model to create a comprehensive approach to a real-world system for categorizing major malicious from a few training instances using computational power with constrained resources. Consequently, the proposed IADM can detect any changes in the IoT networks with a relatively limited number of resources. With a fast and easy detection technique for zero-day attacks, it may improve IDS by making it a fast and easy detection technique with high performance rates.

The future work is to enhance the auto-adaptive model using deep learning and implement more comparative studies to overcome the drawbacks of weak classification algorithms such as J48. Moreover, the enhancement of the processing time of k-NN will be considered.

## Figures and Tables

**Table 1:** Gap analysis of the IADM-based one-shot learning and other proposed models.

Proposed Model	Methodology	Multiple Classification (MC)/Binary Classification (BC)	Auto-Adaptively Based on a Few Numbers of Instances	IoT Scalability Support	Performance Metrics
[1] M. M. Rashid et al., 2020	Machine learning algorithms	MC	×	×	AC, precision, recall, and F1-score: approximately 95%, 94%, and 94%, respectively.
[4] C. Liang et al., 2020	Multi-module system and blockchain using reinforcement algorithms	BC	×	×	AC, precision, recall, and F-scores of 83.9%, 85.53%, 84.14%, and 83.94%, respectively.
[7] I. Alrashdi et al., 2019. AD-IoT	Machine learning algorithm	BC	×	×	AC, false positive rate, and DR: 99.34%, 2%, and 82%, respectively.
[9] E. Anthi et al., 2019	Machine learning algorithms	BC	×	×	Precision, F-measure, and recall: 96.2%, 90.0%, and 98.0%, respectively.
[10] Q. A. Al-Haija and S. Zein-Sabatto, 2020. IoT-IDCS-CNN	Deep learning	BC and MC	×	×	AC of the binary and multi-class classifications of 99.3% and 98.2%, respectively.
IADM	Machine learning techniques and deep one-shot learning	MC	Support auto-adaptivity by applying one-shot learning	Support the scalability manner of IoT	AC, error, precision, recall, F-measure: 98.8%. 2.4%, 97%, and 97%, respectively.

**Table 2:** The correlation ratio of the selected features of the UNSW-NB15 dataset.

Attributes	Correlation	Attributes	Correlation
ct_dst_sport_ltm	80.35%	is_sm_ips_ports	59.04%
ct_src_dport_ltm	71.01%	ct_ftp_cmd	57.6%
ct_dst_src_ltm	69.95%	Service	56.3%
ct_srv_dst	67.34%	response_body_len	55.02%
ct_srv_src	66.83%	Sbytes	54.9%
ct_dst_ltm	64.90%	Rate	52.7%
Id	63.7%	Sttl	51.7%
is_ftp_login	60.9%		

**Table 3:** Performance evaluation of the UNS-NB15 dataset using different machine learning algorithms.

The Performance Metrics	RFT	K-NN	J48	AdaBoost	Bagging
AC	97.99%	97.99%	85.5%	97.99%	91.4%
Error	2.1%	2.1%	14.5%	2.1%	8.6%
Precision	98%	98%	80.9%	98%	91.6%
Recall	98%	98%	82.5%	98%	91.4%
F-measure	97.5%	97.5%	79.5%	97.5%	91.1%

**Table 4:** Performance evaluation of multi-class detection using RFT.

TP Rate	FP Rate	Precision	Recall	F-Measure	Class
97.9%	2.1%	97.9%	97.9%	97.9%	Analysis
97.5%	2.5%	97.5%	97.5%	97.5%	Backdoor
97.9%	2.1%	97.9%	97.9%	97.9%	DoS
97.9%	2.1%	97.9%	97.9%	97.9%	Exploits
97.9%	2.1%	97.9%	97.9%	97.9%	Fuzzers
97.9%	2.1%	97.9%	97.9%	97.9%	Reconnaissance
97.9%	2.1%	97.9%	97.9%	97.9%	Shellcode
97.9%	2.1%	97.9%	97.9%	97.9%	Worms
97.9%	2.1%	97.9%	97.9%	97.9%	Normal

**Table 5:** Performance evaluation of multi-class detection using K-NN.

TP	FP	Precision	Recall	F-Measure	Class
97.9%	2.1%	97.9%	97.9%	98.9%	Analysis
97.9%	2.1%	97.9%	97.9%	98.9%	Backdoor
97.9%	2.1%	97.9%	97.9%	98.9%	DoS
97.9%	2.1%	97.9%	97.9%	98.9%	Exploits
97.9%	2.1%	97.9%	97.9%	98.9%	Fuzzers
97.9%	2.1%	97.9%	97.9%	98.9%	Reconnaissance
97.9%	2.1%	97.9%	97.9%	98.9%	Shellcode
97.9%	2.1%	97.9%	97.9%	98.9%	Worms
97.9%	2.1%	97.9%	97.9%	98.9%	Normal

**Table 6:** Performance evaluation of multi-class detection using J48.

TP	FP	Precision	Recall	F-Measure	Class
23.4%	14.7%	90.2%	23.4%	37.2%	Analysis
48.8%	11.7%	72.1%	48.8%	58.2%	Backdoor
79%	1.4%	76.2%	70%	14.3%	DoS
97.4%	0.1%	67.4%	97.4%	79.7%	Exploits
89.9%	0.7%	96.3%	89.9%	93.0%	Fuzzers
77.5%	0.3%	95.2%	77.5%	85.4%	Reconnaissance
80.2%	0.2%	82.6%	80.2%	81.4%	Shellcode
58.1%	9.1%	58.1%	69.4%	70.8%	Worms
99.7%	0.2%	99.6%	99.7%	99.6%	Normal

**Table 7:** Performance metrics of different classes in multi-class detection using AdaBoost with J48 base learner.

TP	FP	Precision	Recall	F-Measure	Class
97.9%	2.1%	97.9%	97.9%	97.9%	Analysis
97.9%	2.1%	97.9%	97.9%	97.9%	Backdoor
97.9%	2.1%	97.9%	97.9%	97.9%	DoS
97.9%	2.1%	97.9%	97.9%	97.9%	Exploits
97.9%	2.1%	97.9%	97.9%	97.9%	Fuzzers
97.9%	2.1%	97.9%	97.9%	97.9%	Reconnaissance
97.9%	2.1%	97.9%	97.9%	97.9%	Shellcode
97.9%	2.1%	97.9%	97.9%	97.9%	Worms
97.9%	2.1%	97.9%	98.9%	97.9%	Normal

**Table 8:** Performance metrics of different classes in multi-class detection using Bagging with J48 base learner.

TP	FP	Precision	Recall	F-Measure	Class
49.8%	13.1%	90.2%	49.8%	64.4%	Analysis
67.9%	7.82%	84.0%	67.9%	75.1%	Backdoor
58.4%	11.7%	58.4%	66.5%	64.4%	DoS
95.7%	0.8%	81.8%	95.7%	88.2%	Exploits
93.5%	0.5%	97.0%	93.5%	95.3%	Fuzzers
86.0%	0.3%	96.1%	86.0%	90.7%	Reconnaissance
88.6%	0.1%	90.3%	88.6%	89.4%	Shellcode
60.5%	9.7%	92.9%	60.5%	73.2%	Worms
99.8%	0.1%	99.9%	99.8%	99.8%	Normal

**Table 9:** Time consumption of test models using machine learning algorithms.

Machine Learning Algorithms	RFT	K-NN	J48	AdaBoost	Bagging
Time	1.6 msec.	5.0 msec.	6 msec.	14 msec	37 msec

**Table 10:** ROC area of different classes using RFT, K-NN, J48, AdaBoost, and Bagging.

Class	Algorithms				
	RFT	K-NN	J48	AdaBoost	Bagging
Analysis	98.9%	98.9	36.4%	98.9	83.2%
Backdoor	98.9%	98.9	47.8%	98.9	85.4%
DoS	98.9%	98.9	37.5%	98.9	81.6%
Exploits	98.9%	98.9	83.4%	98.9	96.5%
Fuzzers	98.9%	98.9	94.8%	98.9	99.3%
Recon-Naissance	98.9%	98.9	87.6%	98.9	97.8%
Shellcode	98.9%	98.9	85.3%	98.9	95.6%
Worms	98.9%	98.9	64.7%	98.9	91.7%
Normal	98.9%	98.9	100%	98.9	100%
Weighted Average	98.9%	98.9	86.0%	98.9	96.6%

**Table 11:** Performance evaluation.

Model	Maximal Accumulated Reward	Average Accuracy
Anton Model [64]	12.70	78%
Siamese NN [63]	11.31	76%
Our Model	15.29	87%

## Data Availability

Not applicable.
